# EQCM Analysis of the Insertion Phenomena in a *n*-Doped Poly-Alkyl-Terthiophene With Regioregular Pattern of Substitution

**DOI:** 10.3389/fchem.2021.711426

**Published:** 2021-08-19

**Authors:** Danilo Dini, Elisabetta Salatelli, Franco Decker

**Affiliations:** ^1^ Department of Chemistry, University of Rome “La Sapienza”, Rome, Italy; ^2^ Department of Industrial Chemistry “Toso Montanari”, University of Bologna, Bologna, Italy

**Keywords:** conducting polymers, regioregular polythiophene, EQCM gravimetry, n-doping, electrochemical insertion

## Abstract

In the present work, we have undertaken the study of the *n*-doping process in poly-3,3″-didodecyl-2,2′:5′,2″-terthiophene (poly-33″-DDTT) employing the electrochemical quartz crystal microbalance (EQCM). The present study aims at understanding how cathodic charge in *n*-doped poly-33″-DDTT is compensated. For this purpose, the *in situ* analysis of the variations of the polymeric mass has been considered. Poly-33″-DDTT was obtained as a thin coating onto a metallic substrate *via* the anodic coupling of the corresponding monomer 3,3″-didodecyl-2,2′:5′,2″-terthiophene (33″-DDTT). When subjected to electrochemical *n*-doping in the polarization interval -2.5 ≤ *E*_appl_ ≤ 0 V *vs.* Ag/Ag^+^, the films of poly-33″-DDTT varied their mass according to a mechanism of cations insertion during *n*-doping and cations extraction during polymer neutralization. In fact, the electrochemical doping of polythiophenes requires the accompanying exchange of charged species to maintain the electroneutrality within the structure of the polymer in all states of polarization. At the end of a full electrochemical cycle (consisting of the *n*-doping and the successive neutralization of poly-33″-DDTT), the polymer retains a fraction of the mass acquired during *n*-doping, thus manifesting the phenomena of mass trapping. The combined analysis of electrochemical and microgravimetric data suggests that poly-33″-DDTT in the *n*-doped state undergoes (or electrocatalyzes) uncontrolled electrochemical reactions that are not accompanied by mass variations.

## Introduction

**FIGURE 1:**
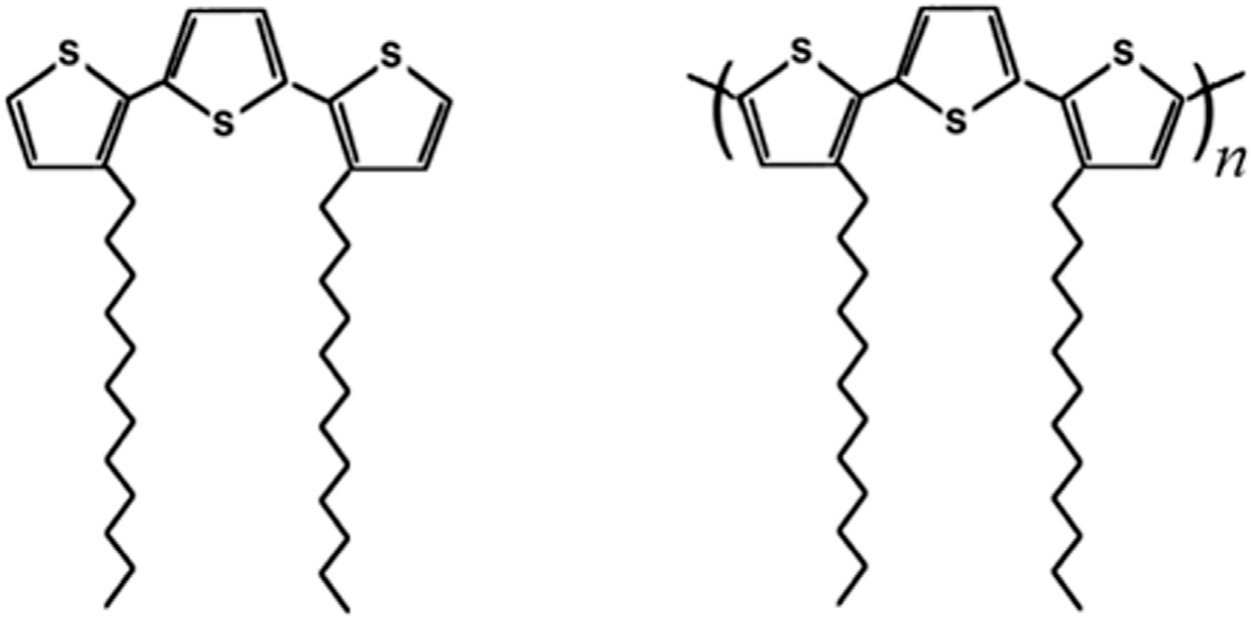
Sketches of 3,3″-DDTT **(monomer on the left)** and poly-3,3″-DDTT **(polymer on the right)**. The monomer 3,3″-DDTT has been employed as a precursor for the electrochemical deposition of the corresponding polymer poly-3,3″-DDTT.

Polythiophenes (PTs) are heterocyclic polymers with an extended area of electronic delocalization (Brédas et al., 2016), which possess high chemical and physical stability (Tourillon and Skotheim, 1986). By virtue of these properties, PTs are successfully employed as active materials in many advanced applications, e.g., photovoltaic cells (Marinelli et al., 2020a; Lanzi et al., 2020; Lanzi et al., 2018; Kippelen et al., 2016), sensors (Wang et al., 2017; Chan et al., 2017a; Guiseppi-Elie et al., 1998), and electroluminescent devices (Kerfoot et al., 2020; Kameta and Shimizu, 2020; Menke et al., 2016), among others (Lodola et al., 2019; Barbarella and Di Maria, 2015; Di Maria et al., 2014; Zessin et al., 2017; Carreon et al., 2014; Chaudhary et al., 2019; da Rocha Rodrigues et al., 2020; Jung et al., 1998; Angiolini et al., 2013; Sheehan et al., 2015; Zheng et al., 2016). PTs are also studied in the ambits of nonlinear optics (Hartmann et al., 2001; Jahja and Bubeck, 2010; Persoons et al., 2016), photonics (Portone et al., 2019; Cornil et al., 1999), and organic electronics (Hsieh et al., 2016; Kanibolotsky et al., 2015; Lee et al., 2016) due to the high polarizability and mobility of the π-electrons present in large concentrations (Xie et al., 2016; Kossmehl and Skotheim, 1986). PTs received great attention among academic and industrial researchers due to the versatility of their synthetic chemistry (Marsitzky et al., 1999; Diaz et al., 1986; Roncali et al., 1998; Alhalasah and Holze, 2005; Roncali, 1997), the diversity of PTs applications (Ellis and Skotheim, 1986; McGehee et al., 1999), and the ability to switch their chemical-physical properties reversibly in response to stimuli of very different nature (electrochemical, electrical, optical, magnetic, thermal, chemical, or biological) (Inganäs, 2010; Otero, 2016). Among various typologies of PTs (Roncali, 1992), there is a general interest in those systems that are derived by the starting monomers constituted by regioregular oligothiophenes (Glenis et al., 1995; Ponnapati et al., 2010; Angiolini et al., 2015). Besides the well-established switching properties that are common in all PTs (Pernites et al., 2011; Xu et al., 2015; Strover et al., 2012; Strover et al., 2017; Friebe et al., 2013; Moreira et al., 2020; Hazra et al., 2020; Chan et al., 2017b; MacDiarmid et al., 1985), this is because the resulting polymers can also display a strong optical activity by virtue of their uncommon chiral properties (Marinelli et al., 2020b; Salatelli et al., 2010). In particular, in this work, we have considered the electrochemical synthesis (MacDiarmid et al., 1985; Biallozor and Kupniewska, 2005; Scrosati, 1988) of the polymer obtained from the anodic coupling of the regioregular monomer 3,3″-didodecyl-2,2′:5′,2″-terthiophene (33″-DDTT; Figure 1, left sketch) (Dini et al., 1998; Dini et al., 1999a; Dini et al., 1999b; Tarola et al., 1999; Tsionsky et al., 1998; Dini et al., 2000) for the analysis of the process of electrochemical n-doping (or, equivalently, the electrochemical reduction). The resulting polymer poly-3,3″-didodecyl-2,2′:5′,2″-terthiophene (poly-33″-DDTT; Figure 1, right sketch) is obtained as a thin film on a supporting metallic substrate when the polymerization of 33″-DDTT monomer (Figure 1, left sketch) is conducted electrochemically through a potentiodynamic route. In this context, we have analyzed the mass variations of poly-33″-DDTT during electrochemical n-doping employing the electrochemical quartz crystal microbalance (EQCM) (Naoi et al., 1991; Buttry and Ward, 1992; Dini et al., 2021), i.e., a non-invasive tool that detects *in situ* the mass changes accompanying a solid-state electrochemical process (Schiavon et al., 1994; Ward and Meyers, 2000). EQCM also represents a sophisticated tool at the basis of those advanced approaches (Gao et al., 2017; Lé et al., 2019) that intend to discern the directions of the fluxes through the electrode/electrolyte interface for the different species involved in an electrochemically driven redox process at an electronically conducting polymer (Arbizzani et al., 1999). In some examples of particular complexity, some researchers have coupled EQCM with AC-electrogravimetry (Lé et al., 2019) and with electroacoustic techniques (Gao et al., 2017) to elucidate the role of anions, cations, and solvent in the charge compensation mechanisms of PEDOT nanowires and dodecylsulfate-doped polypyrrole taking into account the modification of the viscoelastic properties of the polymers undergoing continuous electrochemical cycling. Moreover, EQCM has demonstrated its usefulness in the evaluation of the dynamics of polymerization in the case of the potential pulse sequence-based electrochemical deposition of polypyrrole (Plausinaitis et al., 2015) and the verification of spontaneous adsorption phenomena consisting in the formation of adlayers of pyrrole on metallic substrates prior to any sequence of electrical polarization (Plausinaitis et al., 2017).

Since the electrochemical doping of conducting polymers like PTs is a solid-state reaction (Scrosati and Bruce, 1994) that necessitates the exchange of charged species for the maintenance of electroneutrality in the doped polymer, the use of the EQCM tool appears appropriate in this context (Doblhofer et al., 1998). EQCM analyses of PTs *n*-doping are not frequently reported in the literature (Borjas and Buttry, 1991; Berlin et al., 2004; Casalbore-Miceli et al., 2007; Dini et al., 2021) because the electrochemical reduction of PTs is complicated by the reactivity of PTs in the reduced state in ambient conditions and by the selectivity of reduced PTs towards the charge-compensating cations to be incorporated (Mastragostino and Soddu, 1990; Zotti and Schiavon, 1994; Zotti et al., 1995; Levi et al., 2000). Through this work, we aim to understand the mechanisms of charge compensation/electron injection that underlie the n-doping of poly-33″-DDTT when the process is conducted in a controlled atmosphere taking advantage of the analytical approach we adopted previously (Naoi et al., 1991).

## Experimental Section

### Electrochemical Polymerization

**FIGURE 2:**
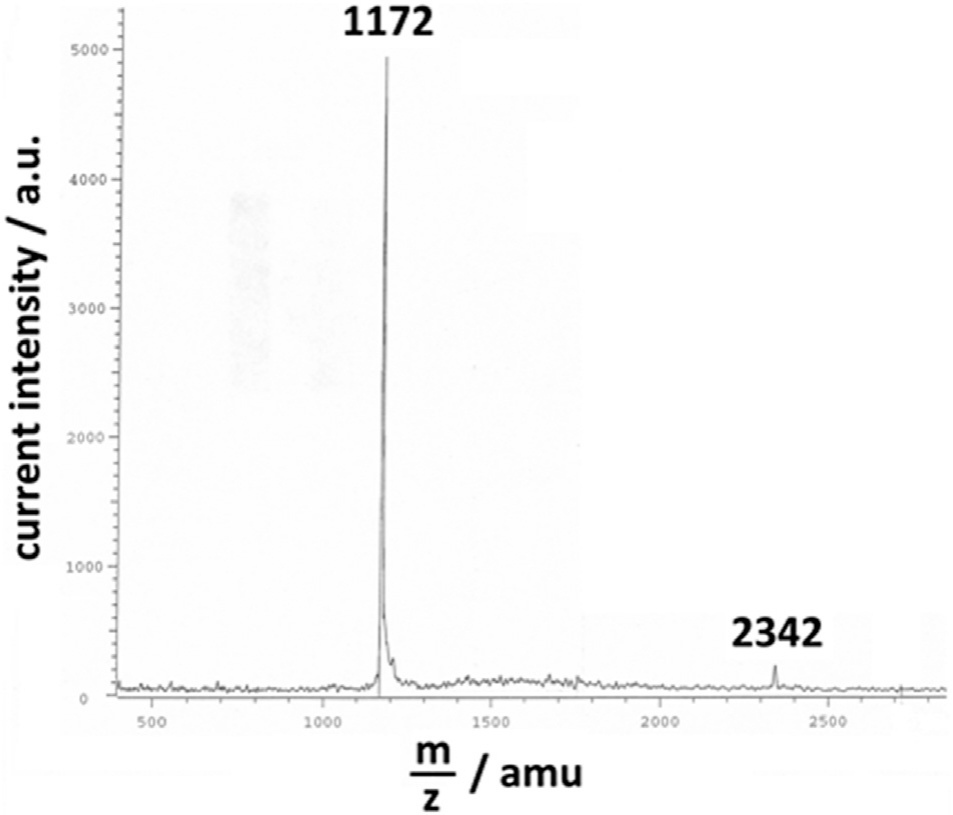
MALDI-TOF spectrum of the fraction of poly-3,3″-DDTT electrodeposit, which is soluble in CHCl_3_. The most intense peaks were detected at 1,172 and 2,342 amu. These values indicate that the CHCl_3_-soluble fraction of poly-3,3″-DDTT was mainly constituted by the dimer and the tetramer of the starting monomer 3,3″-DDTT (with molecular mass 585). In the MALDI-TOF spectrum, the peak associated with the largest mass (not shown) was detected at *m*/*z* = 4,099, i.e., for the oligomer constituted by seven units of 3,3″-DDTT.

**FIGURE 3:**
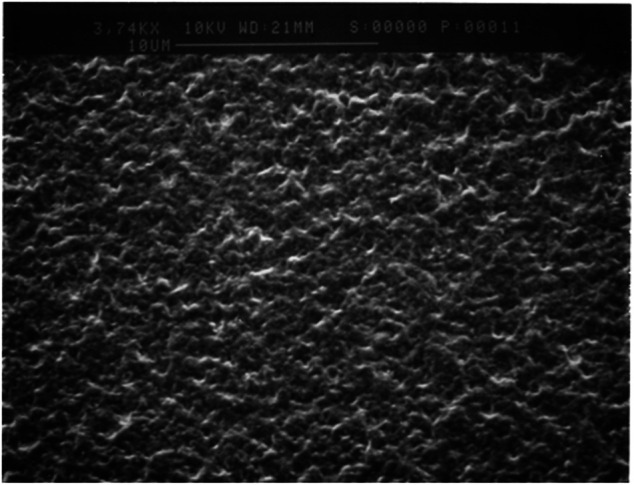
SEM picture of poly-3,3″-DDTT in the neutral state. Similar to the treatment of the electrodeposit described in (Naoi et al., 1991), the polymer was rinsed with CHCl_3_ and successively dried with a stream of N_2_.

For the electrochemical deposition of the corresponding polymer, a solution of electrolysis containing the starting monomer 3,3″-DDTT was prepared. The procedures of electrosynthesis used for the realization of the present paper are very similar to the ones reported in (Naoi et al., 1991), the sole difference being the employment of the isomer 3,3″-DDTT instead of 3′,4′-DDTT. In fact, the solution was obtained by dissolution of the monomer 3,3″-DDTT with variable concentrations (concentration range: 0.3 ≤ *c* ≤ 5 mM) in a mixture of CH_3_CN (acetonitrile, ACN, from Sigma-Aldrich, HPLC gradient grade, ≥ 99.9%) and C_6_H_5_CN (benzonitrile, BN, from Sigma-Aldrich, Reagent Plus grade, 99%). The ACN/BN mixture had the volume ratio of ACN/BN = 4:1 (Dini et al., 1999b; Tarola et al., 1999). ACN and BN were used without any further treatment of purification as in our analogous previous work (Dini et al., 2021). In the solution of electrolysis, the salt (*n*-C_4_H_9_)_4_NClO_4_ (tetrabutylammonium perchlorate, TBAP, from Sigma-Aldrich/Supelco with purity ≥99.0%) was added as supporting electrolyte (SE) at the concentration *c* = 0.1 M. TBAP was dried under vacuum at 70°C before its dissolution in the solution of electrolysis. Prior to the electrochemical deposition of poly-3,3″-DDTT, the solution of electrolysis (containing the monomer and SE) was purged with N_2_ (nitrogen from Air Liquide) for 1 h at room temperature. The electrodeposition of poly-3,3″-DDTT was conducted at room temperature in a three-electrode cell with Ag/Ag^+^ as the reference electrode (RE) [*c*(AgNO_3_) = 0.01 M in ACN] and a Pt wire as the counterelectrode (CE). Pt and Ag wires (diameter, Ø = 1 mm) were purchased from Goodfellow, while AgNO_3_ at the highest purity grade (99.9999%) was obtained from Sigma-Aldrich. The WE of the cell constituted the substrate onto which poly-33″-DDTT was deposited as a thin layer (thickness *l* < 10 μm) (Tsionsky et al., 1998). The choice of the electrode-substrate is conditioned by the type of analysis the polymer film had to pass successively (Dini et al., 1999b; Dini et al., 2021). For the preparation of the polymer sample to be analyzed with the EQCM, cell configuration reported by Naoi et al., 1991, and Persoons et al., 2016, was adopted with an Au-coated AT-cut quartz disk as WE (area 0.35 cm^2^; frequency of resonance 6 MHz) (Dini et al., 1998; Dini et al., 1999a). For the analysis of the UV-Vis properties of poly-3,3″-DDTT, the polymer was deposited onto a conductive substrate made of transparent tin-doped indium oxide (ITO)-covered glass (ITO, from Optics Balzers) with an area of 3 × 1 cm^2^. All polymeric samples were deposited electrochemically in the potentiodynamic mode, the WE being polarized within the applied potential range 0 ≤ *E*_appl_ < 1 V *vs.* Ag/Ag^+^. The potentiodynamic deposition of poly-3,3″-DDTT was conducted at variable scan rates (scan rate range 50 ≤ *v* ≤ 200 mV s^−1^) with supernatant N_2_ in the headspace of the cell. In the chosen range of applied potential, the monomer 3,3″-DDTT and its derived oligomers could be oxidized in order to start the desired process of oxidative polymerization (Tsionsky et al., 1998; Dini et al., 2021). After deposition, the polymer-covered WE was rinsed with CHCl_3_ (chloroform from Sigma-Aldrich with purity ≥99.5%) to remove the polymeric fraction constituted by the shorter chains. The electrodeposit of poly-3,3″-DDTT was not peeled off from the substrate as reported in (Naoi et al., 1991) and remained on the substrate as coating of the WE for the successive experiments of electrochemical *n*-doping. The CHCl_3_-soluble fraction of the electrodeposit contained chains with a maximum degree of polymerization, *n* = 7 (Figure 2), as verified with MALDI-TOF mass spectrometer (instrument from Bruker). This led us to assume that the chains of poly-3,3″-DDTT with *n* ≥ 8, i.e., the fraction insoluble in CHCl_3_, constituted the actual deposit on the WE after its rinsing with CHCl_3_. Before any treatment of electrochemical *n*-doping, the deposit containing the fraction of poly-3,3″-DDTT insoluble in CHCl_3_ was dried under vacuum for 3 h at room temperature and successively kept in a glovebox similar to the procedure reported in (Naoi et al., 1991). The surface morphology of the resulting polymeric sample (Figure 3) was visualized with a scanning electron microscope (SEM), a Cambridge 100 instrument with a resolution of 10 nm when the substrate of polymerization was ITO. The latter was chosen because of its flatness (Dini et al., 2021). The electrochemical polymerization of 3,3″-DDTT was driven by a potentiostat/galvanostat PAR EG&G (mod. 362). The potentiostat was connected to a *Hewlett-Packard* computer through an IEEE-488 GPIB interface similar to what has been reported in (Naoi et al., 1991). Data were acquired and stored in a PC using a LabVIEW Program from National Instruments Corporation as previously reported in (Naoi et al., 1991) and references therein.

**FIGURE 4:**
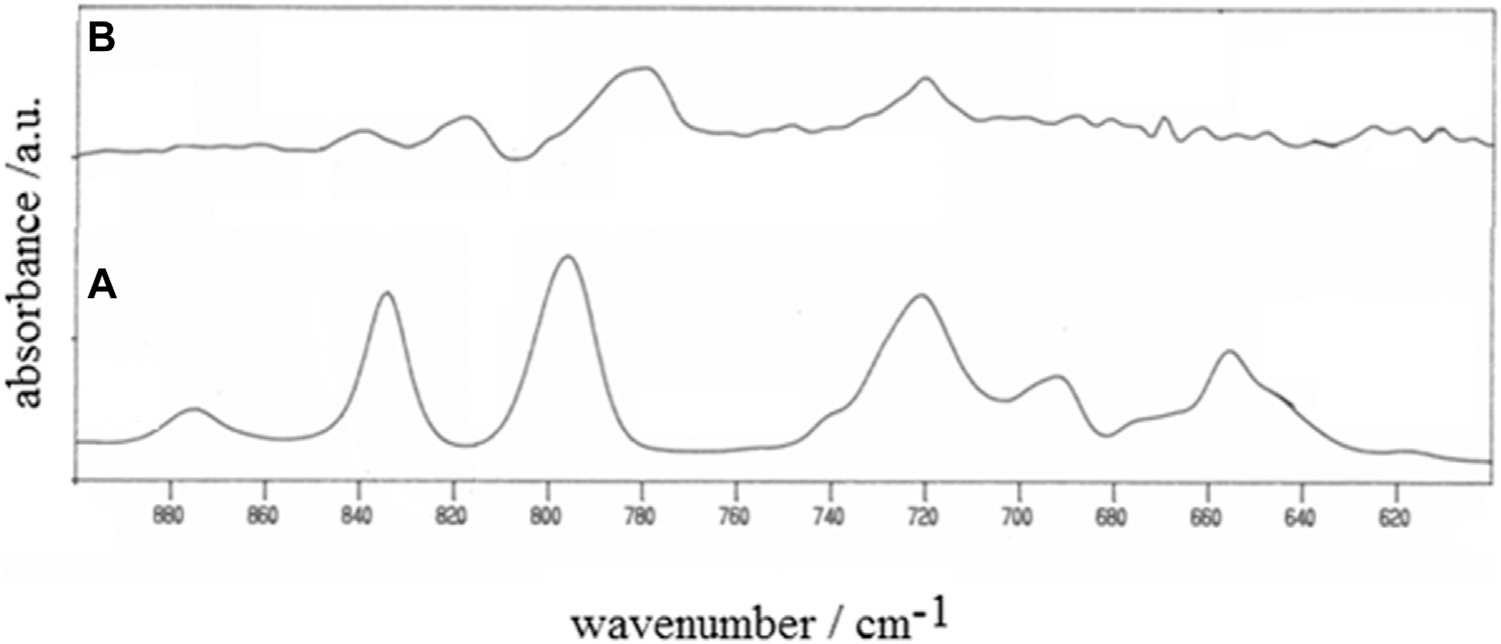
IR spectra of **(A)** monomer 3,3″-didodecyl-2,2′:5′,2″-terthiophene and **(B)** the corresponding electropolymerized product poly-3,3″-DDTT (wavenumber range 600–900 cm^−1^). The polymer was neutral as obtained at the end of the voltammetric experiment of Figure 5 (*vide infra*). The charge of polymer deposition was 25 mC. Spectra were recorded in the reflectance mode.

The occurrence of polymerization was verified *via* IR spectroscopy by comparing the IR profiles of the starting monomer and the electrodeposit (Figure 4). The IR spectra were recorded in the reflectance mode with a Perkin-Elmer System 2000 FTIR employing ITO-coated glass as supporting substrate for both monomer and polymer (Dini et al., 2000). The disappearance of the bands in the range 620–705 cm^−1^ and the attenuation and the shifts of the bands in the range 770–890 cm^−1^ in passing from the monomer to the electrodeposit (Figure 4) are consistent with the occurrence of polymerization during the potentiodynamic oxidation of 3,3″-DDTT (Horak et al., 1966; Hotta et al., 1984; Akimoto et al., 1986; Furukawa et al., 1987).

### Electrochemical n-Doping

Similar to what has been reported in (Naoi et al., 1991), the electrochemical *n*-doping of poly-3,3″-DDTT was conducted potentiodynamically within the applied potential range −2.5 ≤ *E*_appl_ ≤ −0.5 V *vs.* Ag/Ag^+^, using the same electrochemical experimental apparatus already used for the polymerization. The electrolytic solution of polymer doping was a monomer-free 3,3″-DDTT, and it was composed of 0.1 M TBAP in ACN as in the case of the analogous experiments reported in (Naoi et al., 1991). In the n-doping experiments, supernatant N_2_ was flowing in a laminar mode inside the headspace of the cell. In all experiments of polymer *n*-doping, the configuration of the electrochemical cell was a poly-3,3″-DDTT-coated substrate as WE and a Pt wire as CE, while the RE was based on the redox couple Ag/Ag^+^ (0.34 V *vs.* SCE), similar to what has been reported in (Naoi et al., 1991).

### EQCM Analysis

Similar to what has been reported in (Naoi et al., 1991), EQCM was coupled to a homemade oscillator circuit and the oscillation was monitored with a frequency counter from Hewlett-Packard (Model 5316B) (Schiavon et al., 1994). The EQCM was constituted by an Au-coated AT-cut quartz disk as a working electrode with an area of 0.35 cm^2^ and a resonance frequency of 6 MHz (Dini et al., 1998; Dini et al., 1999a). The gravimetric sensitivity (corresponding to the conversion factor of the EQCM) was Δm/Δν = 1.09 ng Hz^−1^ from the calibration experiment of Ag deposition (*vide infra*), and the Sauerbrey equation (Sauerbrey, 1959) was as follows:

$$\frac{\Delta f}{f}=-\frac{\Delta l}{l}=-\frac{\Delta m_{film}}{\rho_{film}A_{sub}l},$$ (1)

where Δ*f* is the vibration frequency variation of the quartz crystal (with characteristic resonance frequency *f*) caused by the deposition of an extra layer of thickness Δ*l* over a layer with initial thickness *l*. In EQCM experiments, the material constituting the extra layer with thickness Δ*l* does not generally correspond with the material constituting the starting layer with thickness *l*, i.e., the Au-coated quartz. Equation 1 shows that for the resonant crystal, the relative change in *f* can be correlated with the mass Δ*m*_*film*_ of the extra deposited layer of poly-33″-DDTT having the thickness Δ*l* and the density *ρ*_*film*_ to the area of the Au-coated quartz substrate (*A*_sub_) provided that the extra layer of the polymeric deposit has the same area of the resonant quartz crystal (absence of border effects) and can be mechanically considered as an extension of the Au-coated quartz substrate (Buttry and Ward, 1992). For the EQCM characterization of poly-33″-DDTT electrochemical *n*-doping reported here, the use of Eq. 1 was justified by the fact that the resonator has a high frequency (>10^5^ Hz) and the working temperature is below the glass transition temperature of the regioregular poly-alkyl-thiophene (>320 K) (Yazawa et al., 2006). Moreover, the frequency change induced by the electrodeposition of poly-33″-DDTT and its n-doping is relatively small with respect to the resonance frequency of the bare substrate. In these conditions, we assume that poly-33″-DDTT film in the neutral and *n*-doped versions is elastic (Buttry and Ward, 1992) and vibrates coherently with the underlying quartz crystal; i.e., the latter is rigidly coupled with the polymer. It is assumed that poly-33″-DDTT (in either the neutral and *n*-doped state) has the same acoustic properties of the quartz crystal when Δ*l* < 350 nm, and it is then treatable with the Sauerbrey equation (Sauerbrey, 1959) in the whole range of applied potential. For the EQCM analysis of electrochemical poly-3,3″-DDTT *n*-doping, the cell was polarized with a potentiostat (mod. 551 from AMEL) modulated by a programmable function generator (mod. 568 from AMEL) similar to the experimental apparatus reported in (Naoi et al., 1991) and references therein. The potential was coupled to a digital integrator (mod. 731 from AMEL) to quantify the electrical charge as in (Naoi et al., 1991). The software analyzing the charge and mass data collected by the computer was homemade (Dini et al., 2021). The software monitored the variations of the vibration frequency in the WE. Moreover, the software correlated the frequency data with WE mass changes by employing the Sauerbrey equation as reported in (Naoi et al., 1991) and references therein. The calibration of the QCM was effectuated by depositing Ag electrochemically from a 10^−2^ M solution of AgNO_3_ in ACN when (C_2_H_5_)_4_NClO_4_ (tetraethylammonium perchlorate, TEAP, Sigma-Aldrich/Supelco with purity ≥99.0%) was the SE in a way similar to what has been reported in (Naoi et al., 1991). The electrode of the QCM is homogeneously covered by the electrodeposit of poly-33″-DDTT when the latter has a thickness within the range of 200–300 nm. In the present study, the thickness of the poly-3,3″-DDTT deposit for the EQCM study ranged between 200 and 350 nm, as verified with an optical profilometer (model WYKO NT1100) (Awais et al., 2013).

### UV-Vis Spectroscopy

The UV-visible absorption spectra of poly-3,3″-DDTT were taken with the experimental apparatus described in (Naoi et al., 1991). This consisted of a Perkin-Elmer spectrometer (mod. Lambda 15) or a diode-array spectrophotometer from HP (mod. 8452A). Spectrophotometers were connected to a computer and driven by software that allowed the recording of data. The software was provided by an instrument supplier similar to what has been reported in (Naoi et al., 1991). The experimental setup is an adaptation of the one reported in refs. (Masetti et al., 1995; Dini et al., 1996), in which we make use of a gas-tight electrochemical cell (Cattarin et al., 1998). For the realization of the n-doping experiments, the cells were assembled under a controlled atmosphere (O_2_ and H_2_O levels lower than 10 ppm) in a glovebox, adopting the same procedure in (Naoi et al., 1991).

## Results and Discussion

**FIGURE 5:**
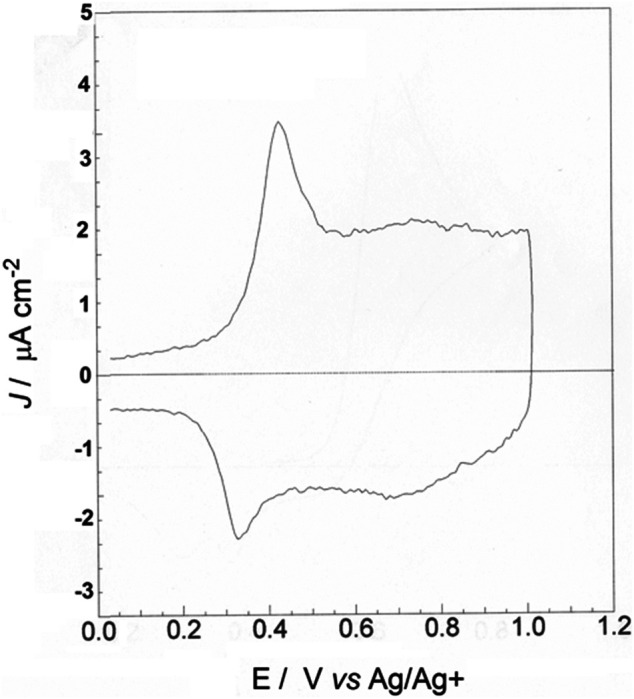
Cyclic voltammetry of poly-3,3″-DDTT deposited onto ITO (scan rate: 100 mV s^−1^; electrolyte composition: 0.1 M TBAP in anhydrous ACN) when the electrodeposit displays a stabilized electrochemical behavior. The deposited charge of poly-3,3″-DDTT was 25 mC. The amount of charge exchanged reversibly by poly-3,3″-DDTT during oxidation was 4 mC.

After rinsing with CHCl_3_ and drying, the electrodeposit of poly-33″-DDTT was electrochemically cycled in the potential interval 0.0 ≤ *E*_appl_ ≤ 1.0 V *vs.* Ag/Ag^+^ (Figure 5). The chosen range corresponds to the one in which poly-3,3″-DDTT is oxidized (Dini et al., 1999b; Tsionsky et al., 1998). The initial potentiodynamic oxidation of poly-3,3″-DDTT was conducted to check its electrochemical behavior and evaluate the characteristics of electronic conjugation. Poly-3,3″-DDTT layer displayed one reversible peak of oxidation at 0.40 V *vs.* Ag/Ag^+^, being this single peak emergent from a broad current wave of capacitive nature. (Ponder et al., 2016). The capacitive current is quasi-constant (*ca*. 2 μA cm^−2^ at the scan rate of 100 mV s^−1^) up to 1.00 V *vs.* Ag/Ag^+^, i.e., the upper limit of applied potential for the oxidative voltammetry. The ratio capacitive current to faradic current (I_cap_/I_far_) is *ca*. 1.6 for poly-33″-DDTT undergoing electrochemical oxidation; i.e., poly-33″-DDTT shows a capacitive contribution larger than the faradic one. Such a value of I_cap_/I_far_ indicates that both processes of anion intercalation (related to the initial faradic process of polymer oxidation under the anodic peaks of Figure 5) (Zotti et al., 1993) and double layer formation (related to pseudo-capacitive phenomena within the polymeric slab during redox-charge injection) (Feldberg, 1984; Scotto et al., 2019) are taking place within poly-33″-DDTT. The co-existence of these two different types of charging phenomena in poly-33″-DDTT leads us to suppose that the charge-compensating anions occupy different types of polymeric sites according to the analysis of Tanguy et al*.*, who distinguished the sites in trapping and non-trapping depending on the depth of anion penetration during the different phases of polymer oxidation (Tanguy et al., 1987).

The current profile of poly-3,3″-DDTT during oxidation is characterized then by a single response. As a such, the voltammogram of poly-3,3″-DDTT recalls one of those polythiophenes having a distorted structure, i.e., with the thiophene rings in an initial non-coplanar conformation (Sannicolò et al., 1998). In poly-3,3″-DDTT, the lack of co-planarity for the concatenated heterocycles along the chain axis is ascribed to the steric hindrance generated by the dodecyl groups that face each other in the starting monomer 33″-DDTT (Figure 1, left sketch). Thanks to their extended length, the two long alkyl substituents (linked in the positions three of monomer terminal rings) can also induce a head-to-head interaction (Zotti and Schiavon, 1989) through the central, unsubstituted ring of thiophene. In this context, it is worth recalling that the polymer obtained from the oxidative polymerization of 3′,4′-didodecyl-2,2′:5′,2″-terthiophene (3′,4′-DDTT, an isomer of 3,3″-DDTT) does not show the same type of voltammetric response as poly-3,3″-DDTT (Figure 5) in analogous conditions of electrochemical cycling (Dini et al., 2021). This is because the current profile of poly-3′,4′-DDTT presents two oxidation levels (Salatelli et al., 2016) *vs.* one level of poly-3,3″-DDTT (Figure 5). This finding leads us to suppose that poly-3′,4′-DDTT possesses high structural order (at the intrachain level) and an extended length (along chain axis) on which adjacent thiophene rings have a coplanar conformation (in contrast to poly-3,3″-DDTT, *vide supra*). Similar to other PTs (Roncali, 1992), the single peak of oxidation of poly-3,3″-DDTT is associated with the injection of positive polarons (spin-bearing species) and positive spin-less bipolarons (Dini et al., 1999b). Such an injection occurs in concomitance with the uptake of negative ions having the function of neutralizing the excess of positive charge in the oxidized polymer (Dini et al., 2000; Soavi et al., 2000; Casalbore-Miceli et al., 2007).

Under these circumstances, the process of positive polarons/bipolarons insertion in oxidized poly-3,3″-DDTT provokes a structural/conformational change, consisting in the transformation of a twisted conformation (typical of a polymer in the neutral non-conductive state) into a flat conformation (the quinoid form). The quinoid form is expected to be not particularly extended in poly-3,3″-DDTT given the steric hindrance of the dodecyl groups (*vide supra*) when poly-33″-DDTT is in the oxidized version that contains initially only polarons. The further oxidation of poly-3,3″-DDTT provokes the insertion of bipolarons (representing the charge carriers of poly-3,3″-DDTT in the regime of heavy oxidation), but such an electrochemical process does not induce any structural alteration of the quinoid form (Dini et al., 1999b; Dini et al., 2021; Zotti and Schiavon, 1989). Poly-3,3″-DDTT at the highest level of oxidation (*E*_appl_ = 1.00 V *vs.* Ag/Ag^+^, Figure 5) is swollen and contains the largest amount of charge-compensating anions (ClO_4_^−^ in the present study) (Dini et al., 1998).

It is then expected that the structure of poly-3,3″-DDTT will suffer a maximum of mechanical stress at *E*_appl_ = 1.00 V *vs.* Ag/Ag^+^. (Dini and Decker, 1998). Mechanical stress is unavoidably experienced by electrochemically switched conducting polymers like poly-3,3″-DDTT undergoing cycles of oxidation/neutralization and reduction/neutralization. This is due to the exchange of the charge-compensating ions between the polymer and the electrolyte to preserve polymer electroneutrality. Such an exchange would provoke variations of surface tension at the (deformable) polymer/electrolyte and (rigid) polymer/Au substrate interfaces to vary the charge content in poly-3,3″-DDTT. The main mechanical effect induced by the simultaneous uptake/release of mass and charge is supposed to consist mostly in the variation of poly-3,3″-DDTT film thickness. Thus, the relief of mechanical stress is achieved through a volume change by poly-3,3″-DDTT, the latter being a deformable system. It is believed that the eventual residual mechanical stress that is not attenuated by poly-3,3″-DDTT through its volume variation produces negligible effects on the response of the quartz resonator in the EQCM. In deformable systems like conducting polymers, the tendency of relieving efficaciously the mechanical stress originated by the insertion/addition of molecular species has rendered the use of EQCM advantageous as an analytical tool for the verification of the occurrence of molecular imprinting of uric acid (Plausinaitis et al., 2020) and caffeine (Ratautaite et al., 2015) in polypyrrole-based sensors.

**FIGURE 6:**
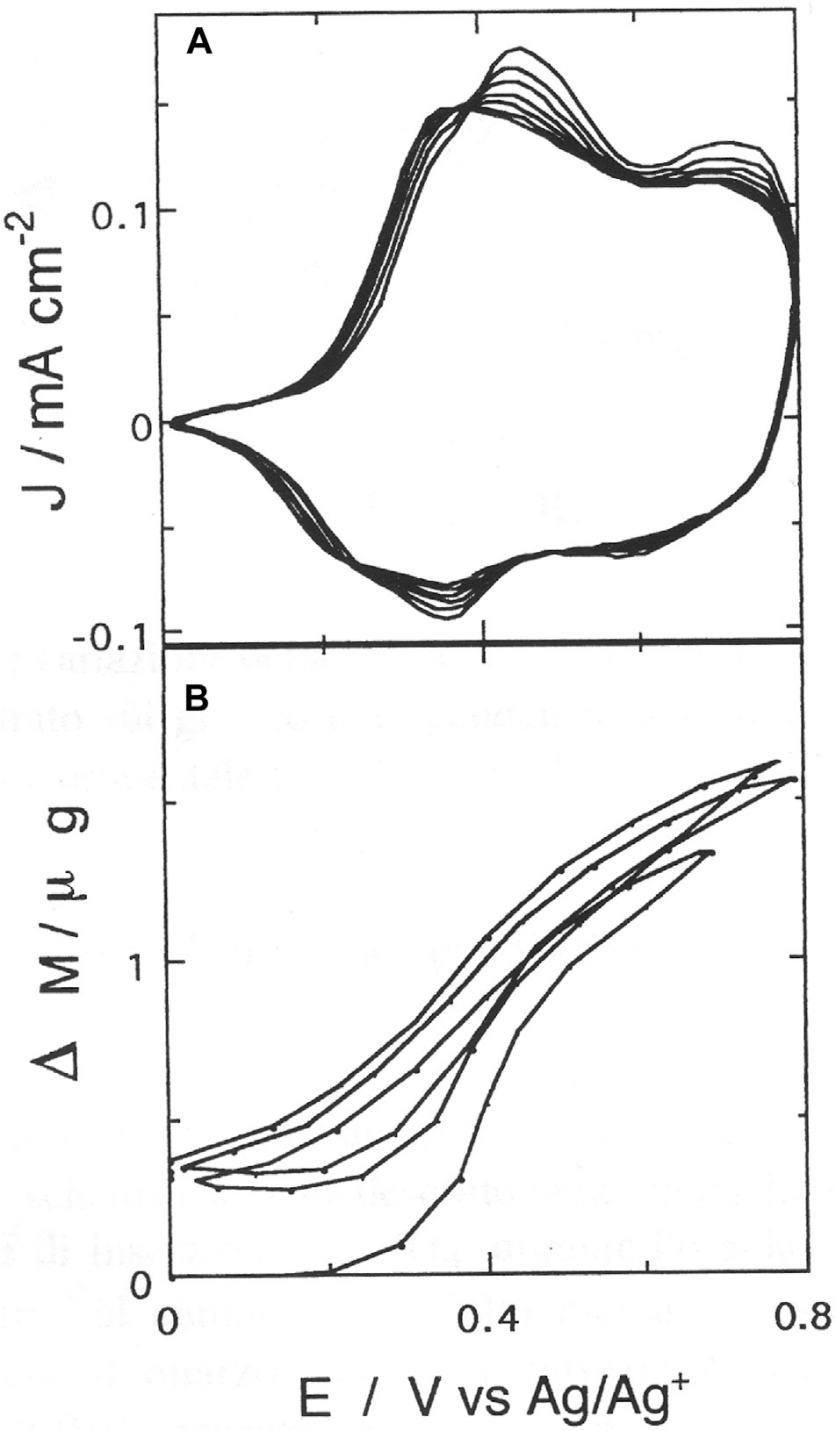
**(A)** Anodic cyclic voltammetry of poly-3,3″-DDTT (scan rate: 50 mV s^−1^); **(B)** electrode mass changes recorded simultaneously *in situ* with EQCM during cyclic voltammetry **(A)**. The mass curve **(B)** shows full reversibility after the first voltammetric cycle at the end of which some mass retention is observed.

In comparison to isomeric poly-3′4′-DDT (Dini et al., 2021), the presence of a relatively sharp cathodic peak in the oxidative voltammogram of poly-3,3″-DDTT denotes the occurrence of a phase transition consisting in a dramatic structural modification of poly-3,3″-DDTT when the extractions of bipolarons (at *E*_appl_ > 0.4 V *vs.* Ag/Ag+) and polarons (at *E*_appl_ < 0.4 V *vs.* Ag/Ag+) take place (Berlin et al., 2004). In poly-3,3″-DDTT, the neutralization of polarons and bipolarons is concomitant with the release of ClO_4_^−^ anions in the adopted experimental conditions. In fact, during the different stages of poly-3,3″-DDTT oxidation, ClO_4_^−^ anions are inserted in the electrochemically oxidized version of the polymer to compensate for its positive charge (Dini et al., 1999b). The absorption spectrum of neutral poly-3,3″-DDTT shows a broad peak with the maximum positioned at 447 nm (not shown). Prior to the EQCM analysis of the electrochemical *n*-doping process in poly-3,3″-DDTT, the polymer was kept at −0.5 V *vs.* Ag/Ag + for 15 min in order to complete its electrical neutralization. The latter process completely removed residual anions from as-deposited poly-3,3″-DDTT (verified with EQCM, *vide infra*). In fact, the EQCM analysis of the electrochemical *p*-doping process in poly-33″-DDTT showed the reversible exchange of the ClO_4_^−^ anion during oxidative-neutralization cycles of poly-33″-DDTT after initial mass retention at the completion of the first voltammetric cycle (Figure 6). Poly-33″-DDTT displayed a stabilized profile of the anodic cyclic voltammogram after 20–25 cycles.

Neutral poly-3,3″-DDTT was dried with a stream of N_2_ at room temperature. After drying, neutral poly-3,3″-DDTT was stored in a glovebox similar to the procedure reported in (Naoi et al., 1991).

### EQCM Characterization of Poly-33′′-DDTT n-Doping

**FIGURE 7:**
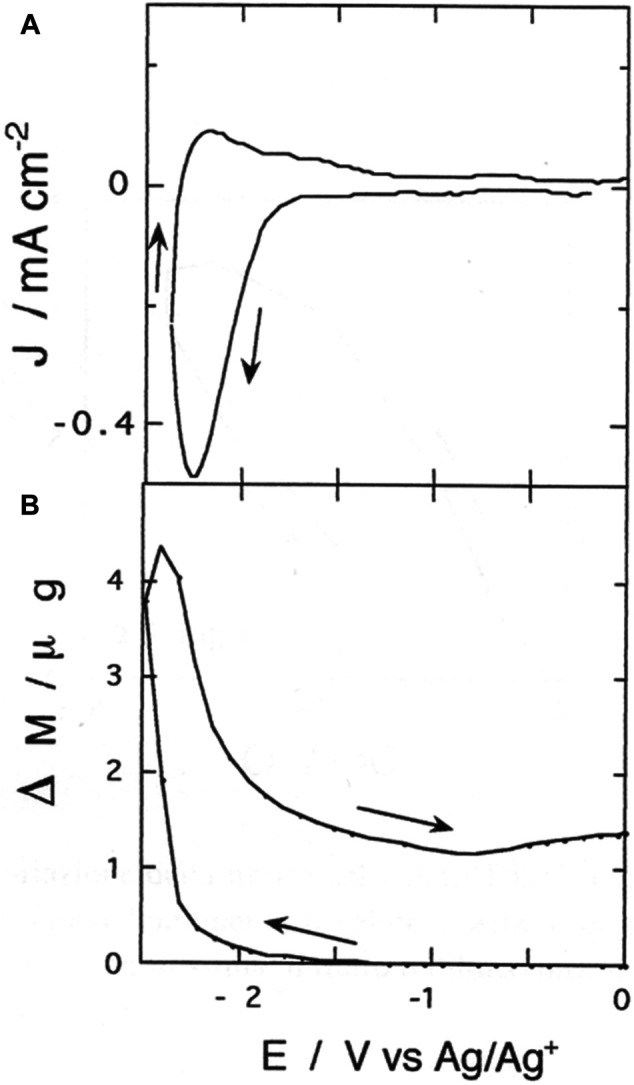
**(A)** Cathodic cyclic voltammetry of poly-3,3″-DDTT (scan rate: 100 mV s^−1^); **(B)** electrode mass changes recorded simultaneously *in situ* with EQCM during cyclic voltammetry **(A)**. Voltammogram **(A)** presents features of irreversibility, while the mass curve **(B)** shows phenomena of mass retention within the polymer at the completion of the applied potential cycle.

The electrochemical reduction of poly-33″-DDTT has the potential onset at *ca*. −1.7 V *vs.* Ag/Ag^+^. The profile of the voltammogram presented a quasi-reversible cathodic peak at −2.24 V *vs.* Ag/Ag^+^ (Figure 7A) when the electrolyte contained TBA^+^, i.e., the organic cation that is expected to play the role of charge-compensating species during *n*-doping (*vide infra*). The reduction voltammogram of Figure 7A has been recorded with a polymer sample thicker than the one employed in the experiment of Figure 4 (80 *vs.* 25 mC of deposited charge). In the reverse anodic scan (i.e., when the applied potential increases from −2.5 to 0 V *vs.* Ag/Ag^+^), the neutralization of reduced poly-33″-DDTT occurs in a single step since it is associated with a singly-peaked current wave of considerably lower intensity in comparison to the reduction peak emerging in the direct cathodic scan (i.e., when potential decreases from 0 to −2.5 V *vs.* Ag/Ag^+^). The important difference between the cathodic and the anodic current of poly-33″-DDTT holds for about ten cycles of potentiodynamic reduction/neutralization. Such an irreversible feature is reproducible during these first cycles. The repeatability of the profile of Figure 6 ceases after *ca*. 20 cycles with the progressive diminution of the current exchanged by the polymer. Upon further cycling, the evolution of the voltammogram is consistent with the degradation of poly-33″-DDTT electrochemical properties and its definitive deactivation.

The associated EQCM response (Figure 7B) is characterized by the increase of polymer mass in correspondence with the onset of the faradic process of poly-33″-DDTT *n*-doping (starting at about −1.7 V *vs.* Ag/Ag^+^). Electrode mass keeps growing up to the maximum levels of polymer reduction (for *E*_appl_ < −2.20 V *vs.* Ag/Ag^+^) and starts to decrease in the reverse anodic scan till it reaches a stationary value for *E*_appl_ >—1 V *vs.* Ag/Ag^+^ (Figure 7B). Such a value is larger than the initial one recorded before starting the electrochemical reductive cycling. Therefore, upon completion of the cycle, poly-33″-DDTT thin film retains an excess of mass (*ca*. 1.5 μg in excess) with respect to the starting state. In doing so, poly-33″-DDTT exhibits an irreversible electrogravimetric behavior with the mass in excess corresponding to *ca*. one-fourth of the total mass gained during *n*-doping by poly33″-DDTT. Different from the complicated electrogravimetric behavior of isomeric poly-3′4′-DDTT undergoing *n*-doping (Dini et al., 2021), the cathodic polarization of poly-33″-DDTT does not induce any variation of electrode mass in the absence of a faradic process.

**FIGURE 8:**
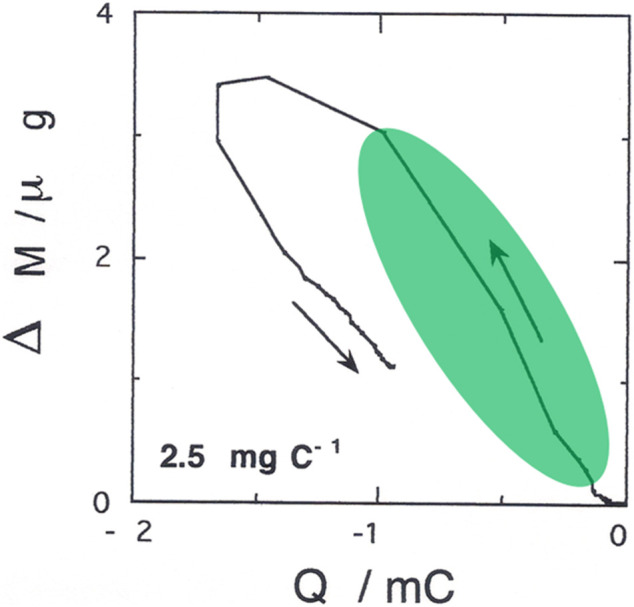
Electrode mass variation (Δ*m*) with the charge (*Q*) exchanged during the first cycle of poly-3,3″-DDTT electrochemical *n*-doping. The applied potential range was −2.5 ≤ *E*_appl_ ≤ 0 V *vs.* Ag/Ag^+^ similar to what has been reported in (Naoi et al., 1991). The value of 2.5 mg C^−1^ in the inset represents the slope of the linear portion evidenced in light green on the curve.

Polymer mass varies with the injected cathodic charge according to the profile of Figure 8. Through this experiment, we aim at identifying the species exchanged by poly-3,3″-DDTT during electrochemical *n*-doping. The time integration of the electrical current profile of Figure 5 allows the calculation of the consumed charge. The analysis of the Δ*m vs. Q* profile reveals that the maximum slope of the approximately linear portion (evidenced in light green in Figure 8) is 2.5*10^-3^ g C^−1^.

This value corresponds to the exchange of a species with a molecular mass of 241 amu when the species bears one positive charge. The mass of the molecular cation TBA^+^ is 242.26 amu, i.e., a value practically coincident with the one determined by the evaluation of the slope. For this reason, we assign to TBA^+^ the role of the actual charge-compensating species for the neutralization of reduced poly-33″-DDTT. Moreover, TBA^+^ constitutes the sole species with a positive charge that can compensate for the excess negative charge in reduced poly-33″-DDTT under the adopted experimental conditions. When *n*-doped poly-33″-DDTT undergoes neutralization, i.e., the inverse process of removal of negative charge from the polymer, a concomitant release of TBA^+^ cations occurs. The release of cations is relatively slow during poly-33″-DDTT neutralization with respect to the extraction of the negative charge from *n*-doped poly-33″-DDTT. This might result in a temporary excess of positive charge within a matrix of an electronically neutral polymer after completely removing the negative charge from poly-33″-DDTT. Such an excess of trapped cations would attract ClO_4_^−^ anions, i.e., the sole mobile species with a negative charge in the adopted experimental conditions. This has no practical consequences on the polymer mass since the migration of ClO_4_^−^ anions towards positively charged poly-33″-DDTT (as a consequence of cations trapping) does not imply the uptake of these anionic species inside the polymer itself, but only the formation of a double layer-like structure at the polymer/electrolyte interface. At the completion of the first cycle, poly-33″-DDTT does not recover the initial value of mass. Consequently, at the start of the second cycle, poly-33″-DDTT still contains TBA^+^, albeit the polymer is in an electronically neutral state. The polymer is then totally deprived of electronic charge carriers (polarons or bipolarons). The second voltammetric cycle (see Supplementary Figure SA1 in the appendix) presents the same gravimetric profile of the first one, the sole difference being the position of the starting point that is upshifted in the second cycle of about 1.2 μg with respect to the initial zero-mass point of the first gravimetric cycle. The (partial) release of TBA^+^ cations occurs concomitantly with the loss of polymeric negative charge in the anodic scan (the linear part with the downward arrow), but there is a region of potential during which mass does not vary linearly with charge. The flattening of the electrogravimetric curve corresponds with potential switching that inverts the scan verse from cathodic to anodic. This finding recalls what we already observed in analogous conditions during the recording of the electrogravimetric curve of isomeric poly-3′4′-DDTT.[**66**] The common electrogravimetric behavior of poly-DDTTs during their electrochemical reduction can be ascribed to parasitic reactions (either electrochemical or electrochemically induced). Possible electrochemical reactions that can be induced by the *n*-switching of poly-DDTTs are water and/or molecular oxygen reduction. In fact, both H_2_O and O_2_ can be present as impurities in the electrolyte. The parasitic reactions of H_2_O and O_2_ reduction would occur simultaneously with the electrochemical reduction of the polymer. On the other hand, H_2_O and O_2_ reductions do not lead to any variation of the electrodic mass. As reported in a previous paper dealing with the analysis of an analogous situation when the isomeric polymer poly-3′4′-DDTT is studied (Dini et al., 2021), the *n*-doping of poly-33″-DDTT constitutes the sole faradic process that can vary the mass of the electrode, and the parasitic reaction does not. Poly-3′4′-DDTT *n*-doping and the parasitic electrochemical reactions occur simultaneously. In this regard, we have to clarify whether the parasitic reactions are directly caused by the negative polarization of the electrode (regardless of the presence of the polymeric layer) or indirectly by the polymer when the latter is brought in the *n*-switched state, i.e., in a hypothetically conductive, electrocatalytic state. In the absence of polymer coating, the bare electrode of the EQCM does not produce any redox current in the range of applied potential where poly-33″-DDTT is electrochemically reduced. Moreover, the blank experiment does not reveal any change of the mass of the bare EQCM electrode; that is, no deposition/adsorption processes are occurring during the cathodic polarization of the bare EQCM substrate. The findings of the blank experiment lead us to conclude that the parasitic reactions are of electrocatalytic nature since they require the presence of reduced poly-33″-DDTT to take place. In this context, reduced poly-33″-DDTT would constitute the actual electrocatalyst of the parasitic reactions. Different from polymer *n*-doping (involving the exchange of TBA^+^), the parasitic reactions do not involve any exchange of species with the polymeric electrocatalyst and, consequently, do not vary the electrode mass. Therefore, the simultaneous occurrence of *n*-doping and parasitic reaction(s) will vary and diminish the slope of the Δ*m vs. Q* curve (effect of curve flattening) with respect to the exclusive occurrence of polymer *n*-doping. In the latter type of process, as a matter of fact, the consumption of charge would be accompanied in parallel by an increase of mass (TBA^+^ uptake) with the generation of a Δ*m vs. Q* curve having a constant, non-zero slope. Different from poly-3′4′-DDTT, the occurrence of parasitic faradic reactions in poly-33″-DDTT leads to a deviation of the slope from the “correct” value of 2.5*10^−3^ g C^−1^ only in a limited range of applied potential (i.e., in correspondence with scan verse switching). Under these circumstances, the passage of negative charge in the range −1.6 ≤ *Q* ≤ −1 mC (Figure 7) cannot be associated exclusively with the exchange of TBA^+^ cations, and a second (unknown) faradic process has to be considered. Identifying the unwanted faradic process goes beyond the scopes of the present article and will require a further analytical study entirely dedicated to this specific aspect.

## Conclusion

Poly-3,3″-DDTT has been obtained as a thin film onto an electrically conducting substrate *via* the electrochemical oxidation of the corresponding terthiophenic monomer when cyclic potentiodynamic conditions were applied. During the process of poly-3,3″-DDTT *n*-doping, the EQCM detected variations in the mass of the polymer-modified electrode, which were consistent with the uptake of TBA^+^ cation for compensating the excess of negative charge in reduced poly-3,3″-DDTT. This is because the analysis of a Δ*m vs. Q* curve led to the determination of 2.5 mg C^−1^ as the slope of the linear portion. The value of 2.5 mg C^−1^ would correspond to the exchange of a species with a molecular mass of 241 amu, when the exchanged species carries one positive charge (TBA^+^ has a mass of 242 amu). Upon reversal of the potential, i.e., in the phase of polymer neutralization, poly-3,3″-DDTT does not recover its initial mass, thus manifesting phenomena of charge retention at the completion of a voltammetric cycle. It is supposed that the excess of positive charge retained by poly-3,3″-DDTT will recall negatively charged counterions. The latter, in turn, will not be adsorbed on the polymer surface since no increase of electrode mass could be detected during polymer neutralization in the anodic scan. Similar to what has been reported in (Naoi et al., 1991), when the applied potential approaches the lower limit of −2.5 V *vs.* Ag/Ag^+^, poly-3,3″-DDTT in the *n*-switched state might act as an electrocatalyst towards parasitic reactions that occur either sequentially or in parallel to poly-3,3″-DDTT *n*-doping. These uncontrolled electrochemical reactions consume charges but do not alter the electrode mass. This led us to conclude that the parasitic reactions are not solid-state electrochemical processes (Scrosati and Bruce, 1994). These unwanted and uncontrolled electrochemical reactions cause the generation of a voltammogram with irreversible features and are accompanied by the exchange of ca. 0.6 mC of cathodic charge. Such cathodic reactions are mass transport-limited (for the presence of a current peak) and are expected to be electrocatalyzed by poly-3,3″-DDTT in the reduced n-doped state (*vide supra*). In conclusion, poly-3,3″-DDTT undergoing n-doping/undoping cycles presented irreversible features in terms of electrical current and mass exchange. Different from other similar polymeric conducting systems (Dini et al., 2021), in poly-3,3″-DDTT, the kinetics of cation uptake during the cathodic scan was the same as that in the anodic scan of neutralization. As far as the applicability of poly-33″-DDTT is concerned, the analysis of the electrochemical *n*-doping process here reported demonstrates that this regioregular poly-terthiophene can be exploited directly without molecular decorations as sensing element for the electrochemical detection of cations, provided that such a task is conducted by cathodically polarized poly-33″-DDTT in a non-aqueous environment. Moreover, it is expected that poly-33″-DDTT is generally prone to molecular imprinting with a variety of molecules for the creation of functional structures having high binding selectivity given the versatility and the mildness of the conditions for the electrochemical synthesis of a poly-thiophene from a terthiophenic monomer.

## Data Availability

The original contributions presented in the study are included in the article/Supplementary Material; further inquiries can be directed to the corresponding author.
