# Supplementary material for: EQCM Analysis of the Insertion Phenomena in a n-Doped Poly-Alkyl-Terthiophene With Regioregular Pattern of Substitution
Source: Front Chem. 2021 Aug 19;9:711426. doi: 10.3389/fchem.2021.711426 (PMC8417062; doi:10.3389/fchem.2021.711426)
Supplement: Supplementary file 1 [file DataSheet1.pdf]

Appendix

Title: EQCM analysis of the insertion phenomena in a *n*-doped poly-alkyl-terthiophene with regioregular pattern of substitution

Authors: D. Dini, E. Salatelli, F. Decker

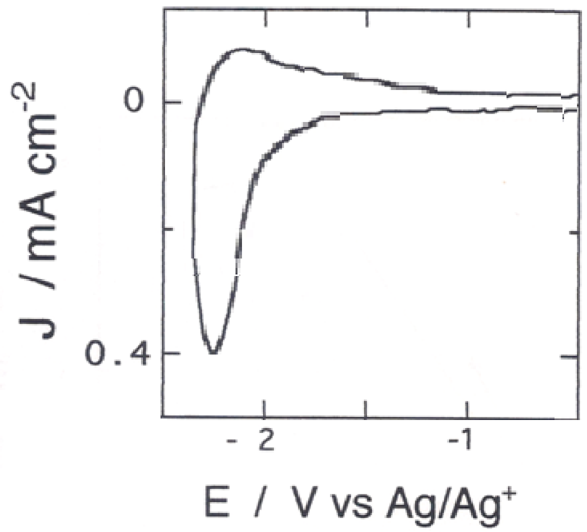

Figure A1: Second cathodic cyclic voltammetry of poly-3,3″-DDTT at the scan rate of 100 mV s^{-1}. Similar to the first cycle (Figure 6a) there is the observation of a considerable difference between the cathodic current and the anodic current.
